# The Collothecidae (Rotifera, Collothecacea) of Thailand, with the description of a new species and an illustrated key to the Southeast Asian fauna

**DOI:** 10.3897/zookeys.315.5330

**Published:** 2013-07-04

**Authors:** Phuripong Meksuwan, Pornsilp Pholpunthin, Hendrik Segers

**Affiliations:** 1 Plankton Research Unit, Department of Biology, Faculty of Science, Prince of Songkla University, Hat Yai 90112, Songkhla, Thailand; 2 Freshwater Laboratory, Royal Belgian Institute of Natural Sciences, Vautierstraat 29, 1000 Brussels, Belgium

**Keywords:** Diversity, identification key, sessile rotifers, Southeast Asia, uncinate trophi

## Abstract

Following previous reports indicating a remarkable high diversity of sessile rotifers in Southeast Asian freshwaters, we report on an extensive study of the diversity of Collothecidae rotifers from fifteen freshwater habitats in Thailand. A total of 13 species, including two additional infraspecific variants, of Collothecidae are recorded, one of which is described as a new species of *Collotheca*. We further add taxonomic remarks on some of the taxa on record and illustrate the uncinate trophi of several representatives by scanning electron microscopic images. Finally, we provide illustrated identification keys to the Collothecidae recorded to date from Southeast Asia.

## Introduction

Family Collothecidae is one of two families of the rotifer Order Collothecacea. The order is diagnosed by the presence of uncinate trophi (Segers 2002, Wallace et al. 2006) and a peri-buccal region expanded into a wide infundibulum, while family Collothecidae is further characterized by having a modified *corona ciliata* (short: corona) consisting of differentiated cilia implanted along the margin of, or grouped on knob-like, lobate or tentacle-like extensions of the infundibulum. The family contains two genera, *Collotheca* Harring and *Stephanoceros* Ehrenberg, and these respectively contain 45 and one valid species (Koste 1978, Segers 2007). Collothecid rotifers are essentially ambush predators. Their expanded and elongated corona lobes and cilia lobes form a fyke-like structure by which mobile prey, either zoo- or phytoplankton, are directed towards an enlarged funnel-shaped infundibulum. Once there, prey is trapped by contraction of infundibular sphincter muscles and swallowed through the pumping action of a membrane supported by the rod-shaped trophi. This specialized feeding strategy and its phylogenetic consequence have received considerable attention by rotifer research (e.g., Kutikova and Markevich 1993, Sørensen and Giribet 2006), although large gaps remain in our knowledge of the diversity and evolution of the group.

To date, comparatively little is known on the distribution and diversity of sessile rotifers in general and of Collothecidae in particular, which is due to the fact that these animals require life observation for identification and study. This knowledge gap is especially evident regarding sessile rotifers from tropical regions. These animals are mostly dealt with on an *ad hoc* basis, and much of what little information that exists is contained in more general inventories of rotifers, in which the sessile taxa are represented as chance occurrences (e.g., Chittapun et al. 2007, Sanoamuang and Savatenalinton 2001, Segers and Chittapun 2001, Segers and Sanoamuang 2007). Some recent relevant studies on Southeast Asian sessile rotifers (Koste 1975, Meksuwan et al. 2011, Segers et al. 2010) report a remarkable diversity of the group, including several species of outstanding taxonomical and/or biogeographically interest, which sparked a more comprehensive study on this particular taxon of rotifers. Here we report on the diversity and taxonomy of Collothecidae found during our extensive study of the sessile rotifers of Thailand. Finally, realizing that the only available, relatively recent identification work dealing with Collothecidae is in German (Koste 1978), we present a key to the identification of the Collothecidae recorded from Southeast Asia, to facilitate and promote future studies on these remarkable animals.

## Material and methods

**Figure 1.**
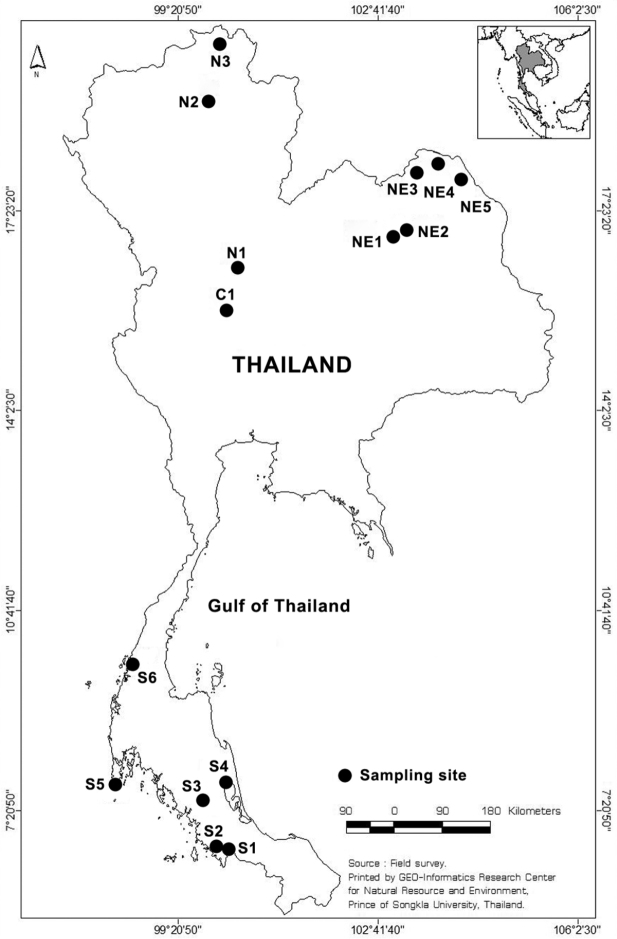
Sampling sites in Thailand. S, C, N and NE represent sampling sites in the Southern, Central, North and Northeast part of Thailand, respectively. Map from GIS center, PSU.

We explored 15 freshwater habitats in 12 provinces of Thailand for Collothecidae during the present study (Fig. 1). Submerged parts of different species of aquatic plant were collected qualitatively to search for sessile rotifers. Collecting and observation methods are detailed in Meksuwan et al. (2011). Searching and identifying rotifers was performed under an Olympus SZ 51 stereo microscope and an Olympus CX 21 compound microscope. Drawings are based on photographs and observations of living animals. Trophi were prepared for scanning electron microscopy (SEM) following the method of Segers (1993), SEM photographs were taken using a FEI Quanta 400 SEM at the Scientific Equipment Center, Prince of Songkla University, Hatyai campus.

## Results and discussion

### Diversity of family Collothecidae in Thailand

The samples examined contained 13 species and two infraspecific variants of Collothecidae (Table 1). This corresponds with ca. 28% of the world fauna of *Collotheca* species and all *Stephanoceros* species known to date (Segers 2007). Two of the species identified could not be ascribed to any known species and we conclude that the specimens pertain to new species, one of which is described below. Of the second possibly new species we opine that insufficient material is at present available to warrant a full description, hence we only provide a brief illustration to enable future recognition. One more species, *Collotheca ferox* (Penard) is new to the Oriental region and *Collotheca ornata* f. *cornuta* (Dobie) is new to Thailand. These results indicate a relatively diverse Collothecidae fauna in the studied region of Thailand, and the record of one, and possibly two new species leads us to surmise that an even higher and incompletely documented diversity can be expected to occur in Southeast Asia.

**Table 1.** List of Collothecidae species recorded from Thailand<br/>

Family Collothecidae Harring, 1913
Genus *Collotheca* Harring, 1913
*Collotheca algicola* (Hudson, 1886)
*Collotheca ambigua* (Hudson, 1883)
*Collotheca campanulata* (Dobie, 1849) (incl. f. *longicaudata* (Hudson, 1883)
*Collotheca edentata* (Collins, 1872)^1^
*Collotheca ferox* (Penard, 1914)*
*Collotheca heptabrachiata* (Schoch, 1869)
*Collotheca orchidacea* sp. n.*
*Collotheca ornata* (Ehrenberg, 1832) (incl. f. *cornuta* (Dobie, 1849)**)
*Collotheca stephanochaeta* Edmondson, 1936
*Collotheca tenuilobata* (Anderson, 1889)
*Collotheca trilobata* (Collins, 1872)
*Collotheca* sp.
Genus *Stephanoceros* Ehrenberg, 1832
*Stephanoceros fimbriatus* (Goldfusz, 1820)
*Stephanoceros millsii* (Kellicott, 1885)

* = new to Oriental region and Thailand;

** = new to Thailand

^1^recorded by Koste (1975), not seen during this study

## Taxonomy

### Genus *Collotheca* Harring

#### Collotheca ferox

(Penard)

http://species-id.net/wiki/Collotheca_ferox

##### Remarks.

**Figure 2.**
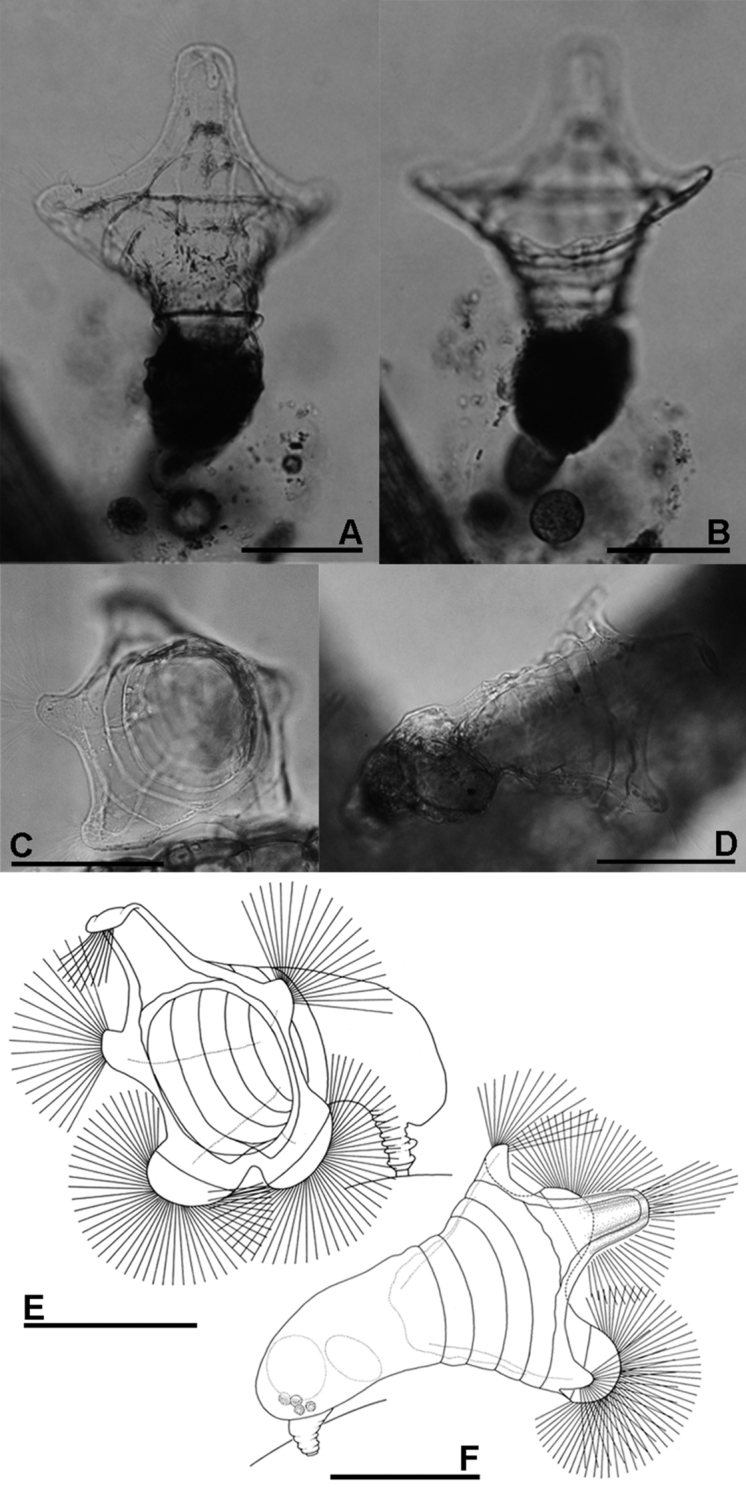
**A, B** *Collotheca ferox* (**A** dorsal view **B** ventral view) **C–F** *Collotheca orchidacea* sp. n. (**C, E** frontal **D, F** dorsal). Scale bars: **A–F** = 100 µm (**A, B** by Rapeepan Jaturapruek).

The morphological characters of our specimens agree closely with the description of the species by Penard (1914): the corona of the specimens is more than twice as broad as its trunk and bears five broad lobes (Fig. 2A, B, 4J). The dorsal lobe tip is relatively large and rounded anteriorly; the lateral lobes are intermediate in size whereas the triangular ventral lobes are relatively small and are set close together. The features of the ventral lobe are unique to this species and prevent confusion with other five-lobed species of the genus. Our photographs of living specimens and trophi of *Collotheca ferox* confirm, in particular, the unique features of the ventral corona lobes illustrated by Penard (1914).

#### Collotheca orchidacea

Meksuwan, Pholpunthin & Segers sp. n.

urn:lsid:zoobank.org:act:E7CA6ECF-175D-4E46-BCA8-970FA4F5C9CC

http://species-id.net/wiki/Collotheca_orchidacea

Figs 2C–F , 5E

##### Type locality.

Thale Noi Lake, Phatthalung Province, Thailand: 7°47.378'N, 100°8.969'E, on *Utricularia* sp., mostly on the surface of the bladder traps, 18 March 2012, P. Meksuwan leg.

##### Type specimens:

Holotype female mounted in permanent microscope slide, in Princess Maha Chakri Sirindhorn NaturalHistory Museum, Prince of Songkla University, Songkhla, Thailand, PSUZC-PK5PM2-1. Original label: “Rotifera, Family Collothecidae, *Collotheca orchidacea* Meksuwan & Segers, Locality: Thale Noi Lake, Phattalung Province, Thailand, Collected by P. Meksuwan 18-3-2012, Holotype”; two paratype females in permanent microscope slides, inRoyal Belgian Institute of Natural Sciences, Brussels, Belgium, IG 32158 RIR 204-205. Original label: “Rotifera, Family Collothecidae, *Collotheca orchidacea* Meksuwan & Segers, Locality: Thale Noi Lake, Phattalung Province, Thailand, Collected by P. Meksuwan 18-3-2012, Paratype”.

##### Differential diagnosis.

The presence of a five-lobed corona separates the new species from most of the known members of genus *Collotheca*. In comparison with other *Collotheca* species having a five-lobed corona (*Collotheca algicola* (Hudson), *Collotheca ambigua* (Hudson), *Collotheca annulata* (Hood), *Collotheca bilfingeri* Bērziņš, *Collotheca ferox* and *Collotheca campanulata* (Dobie)), *Collotheca orchidacea* sp. n. can be distinguished by its uniquely well-developed thumb-shaped lateral and semi-circular ventral corona lobes. It has a relatively broad infundibulum, and short foot and trunk, similar only to *Collotheca ambigua* and *Collotheca ferox*. In addition, *Collotheca orchidacea* sp. n. and *Collotheca ferox* hold their infundibulum and corona towards the substratum, whereas most other species including *Collotheca ambigua* and *Collotheca campanulata* normally hold their body and corona upright.

##### Description.

Habitus (Fig. 2C–F): infundibulum funnel-shaped, trunk and corona opening held horizontally. Infundibulum and proventriculus about twice as long as the trunk. Infundibulum large, more than twice as wide as trunk. Foot short, length about half of trunk, contractile, with a short peduncle. Corona five-lobed: single dorsal, and a pair of well-developed lateral and of ventral lobes. Infundibulum with a weak line running parallel to the edge of the corona, and at least four ring-shaped structures (circular muscles?). Dorsal lobe large, elongate, basally with straight and converging lateral margins; parallel-sided medially, with smoothly curved antero-lateral corners. Tip of dorsal lobe transversally sinuate. Lateral lobes relatively the smallest, thumb-shaped, about half as wide as the dorsal lobe. Ventral lobes broadest, smoothly rounded, separated by a large and deep sinus. A group of setae present on the tip of all corona lobes.

Trophi (Fig. 5E) uncinate. Two pairs of subequal unci teeth relatively equal in length. All arrow head unci with middle groove.

##### Measurements.

Females total length ca. 340 µm. Length of infundibulum plus proventriculus ca. 190 µm, trunk ca. 100 µm, foot ca. 50 µm. Trunk width ca. 70 µm. Infundibulum width ca. 180; dorsal lobe length ca. 75 µm, width ca. 30 µm; ventral lobe width ca. 120 µm, ventral sinus depth ca. 30 µm.

##### Etymology.

The species name – *orchidacea* is a noun in apposition, and refers to the shape of the new species’ corona, which is reminiscent of the flower of certain orchid species. As such, the name of the species also refers to the biodiversity of Thailand, characterized by an abundance of orchid species.

##### Distribution.

The species is known from its type locality only.

#### Collotheca ornata f. cornuta

(Dobie)

##### Note.

This taxon (Figs 4G, H) is differentiated from the nominal form by the corona bearing an elongate projection dorsally to the dorsal lobe. The presence/absence of this projection has classically been interpreted as of infrasubspecific relevance only (see Edmondson 1940, Koste 1978). In the absence of additional data (morphological, molecular or behavioural), we prefer to be cautious and record the taxon separately.

Specimens were found in Khlong Lam Chan Non-Hunting Area, Trang province (Fig. 1: S3); the present is the first Thai record of the taxon.

#### Collotheca sp.?

##### Remarks.

**Figure 3.**
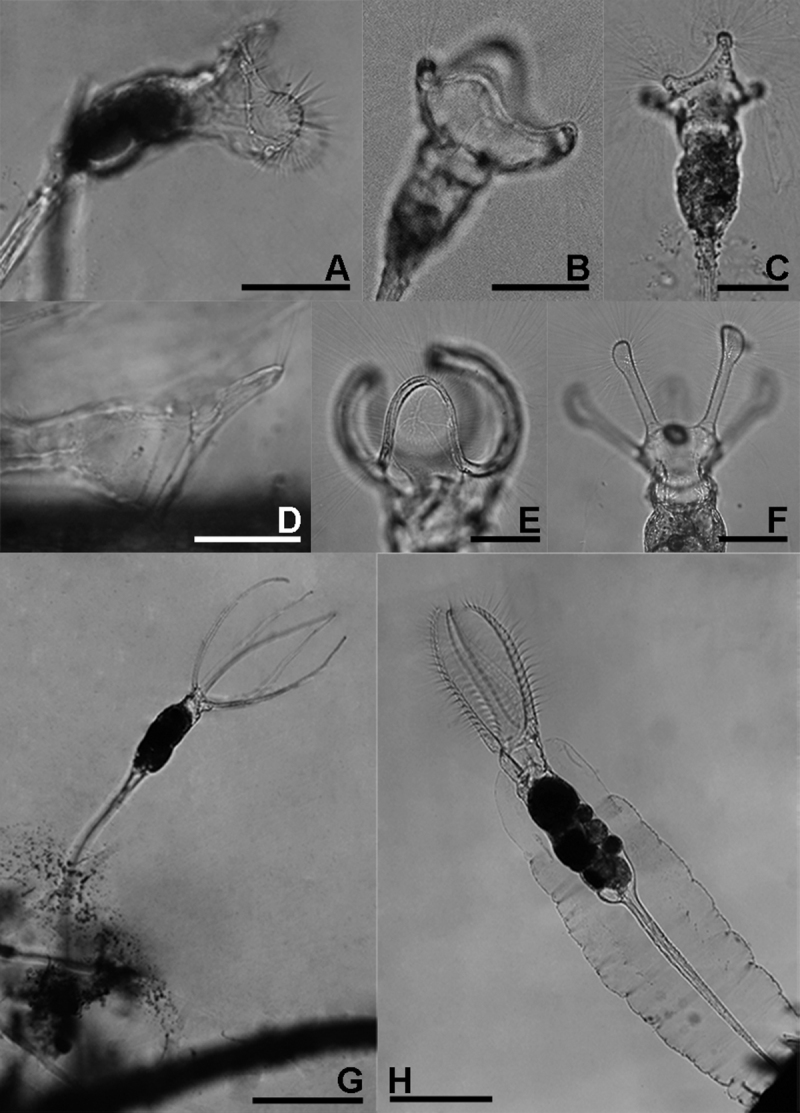
*Collotheca* and *Stephanoceros* species. **A** *Collotheca stephanochaeta*, lateral **B** *Collotheca campanulata* f. *longicaudata*, ventral **C** *Collotheca ornata*, dorsal **D** *Collotheca* spec., lateral **E** *Collotheca trilobata*, lateral **F** *Collotheca tenuilobata*, ventral **G** *Stephanoceros millsii*, lateral **H** *Stephanoceros fimbriatus*, lateral. Scale bars: **B–D** = 50 µm, **A, E, F** = 100 µm, **G, H** = 250 µm.

We found a single specimen of a species that we could not identify (Fig. 3D). Its corona consists of two lobes, one large dorsal lobe and one minute ventral lobe, which is similar to *Collotheca calva* (Hudson). The specimen, however, exhibits a unique cluster of long setae dorsally on the tip of the dorsal lobe and, in addition, shows two ring-shaped structures in the infundibulum. The presence of an egg in its gelatinous case indicates that the specimen was mature and not some incompletely developed juvenile. We believe that it represents an undescribed species but refrain from describing and naming it due to the lack of a sufficient number of specimens. The animal occurred in Khlong Lam Chan Non-Hunting Area, Trang province (Fig. 1: S3).

**Figure 4.**
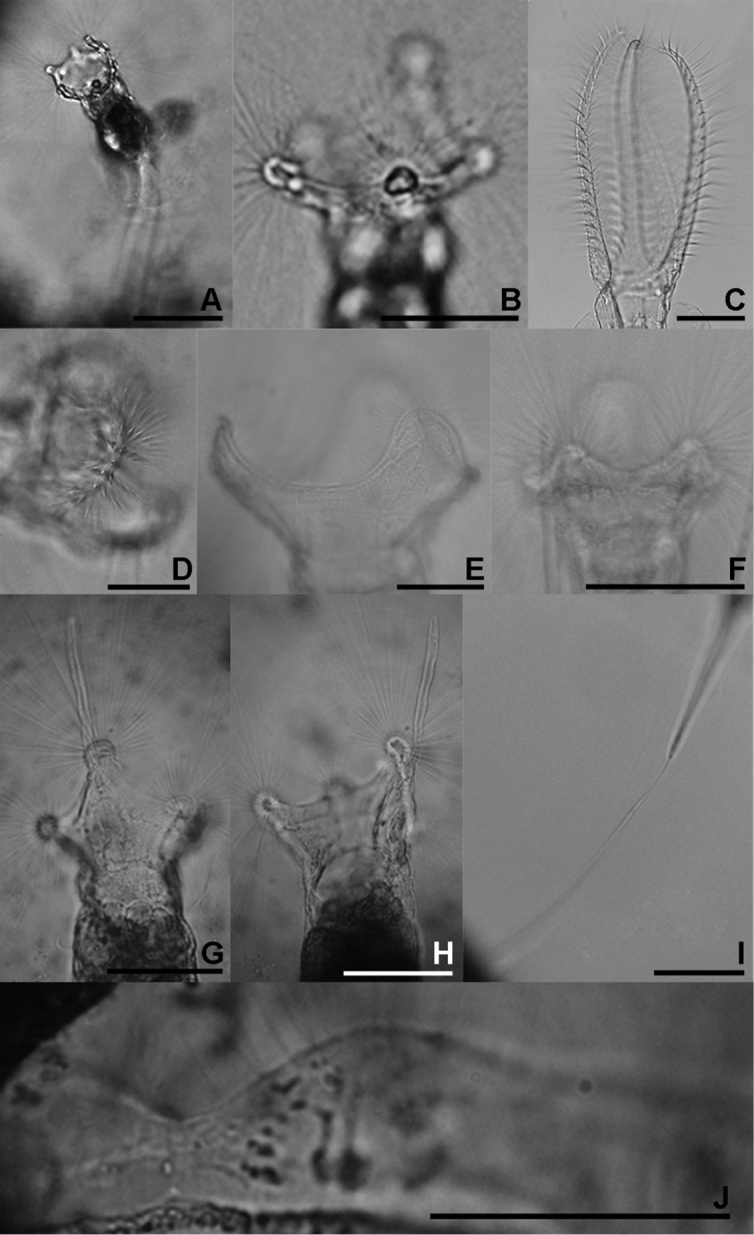
**A** *Collotheca heptabrachiata*, lateral **B** *Collotheca ornata*, ventral **C** *Stephanoceros fimbriatus*, lateral **D** *Collotheca stephanochaeta*, lateral **E** *Collotheca ambigua*, ventral **F** *Collotheca algicola*, ventral **G, H** *Collotheca ornata* f. *cornuta* (**G** dorsal **H** lateral) **I** *Collotheca campanulata* f. *longicaudata*, attachment stalk **J** *Collotheca ferox*, ventral corona margin. Scale bars: **A, B, D–H, J** = 50 µm, **C, I** = 100 µm.

**Figure 5.**
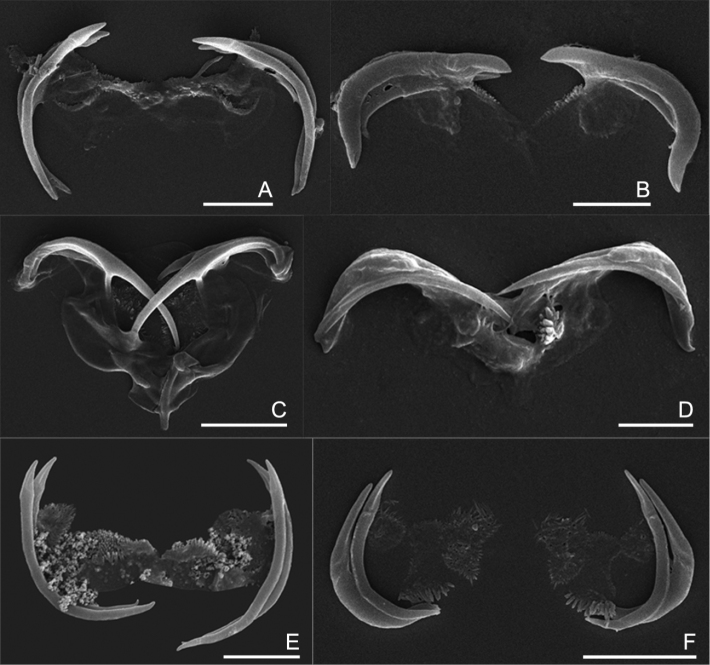
The uncinate trophi of Collothecidae species. **A** *Collotheca ferox* **B** *Collotheca campanulata* **C** *Collotheca trilobata* **D** *Collotheca tenuilobata* **E** *Collotheca orchidacea* sp. n. **F** *Stephanoceros millsii*. Scale bars: **A, B, D, E** = 5 µm, **C, F** = 10 µm.

#### Genus Stephanoceros

Ehrenberg

http://species-id.net/wiki/Stephanoceros

##### Remarks.

Genus *Stephanoceros* is diagnosed (Koste 1978) by having extraordinarily long extensions of the corona (tentacles) bearing transversally implanted rows of medium-long cilia, in addition to short mobile cilia. Following this diagnosis he suggests that *Collotheca stephanochaeta* (Edmondson), which has short corona lobes bearing similarly inserted rows of cilia, might be better placed in *Stephanoceros* rather than *Collotheca*, while he discards the relevance of the absence of such transverse rows of cilia in *Stephanoceros millsii* (Kellicott) by considering the latter a mere infrasubspecific ecotype of *Stephanoceros fimbriatus* (Goldfusz).

We believe that the diagnosis of *Stephanoceros* is questionable, considering that neither the presence of long corona lobes (also in *Collotheca judayi* Edmondson and *Collotheca tenuilobata* (Anderson)) nor the presence of transverse rows of cilia on the corona lobes (present in *Collotheca stephanochaeta*, absent in *Stephanoceros millsii*, see below) can serve as synapomorphic diagnostic feature for the genus. To the contrary, we hypothesize that the two species now attributed to *Stephanoceros* are merely species in which the prolongation of corona lobes already present in many species of *Collotheca* has evolved to its greatest extent. We look forward to a more complete phylogenetic analysis of the taxa, knowing that a molecular phylogenetic study of the group is on-going. A synonymy between *Collotheca* and *Stephanoceros* would have to result in the reallocation of all taxa of the junior *Collotheca* to the senior *Stephanoceros*.

### *Stephanoceros fimbriatus* (Goldfusz) versus *Stephanoceros millsii* (Kellicott) (revised status)

We found specimens matching the descriptions of two taxa in *Stephanoceros*, *Stephanoceros fimbriatus* (Fig. 3H) and *Stephanoceros millsii* (Fig. 3G, 5F) (see Kellicott 1887, Koste 1978). *Stephanoceros fimbriatus* has five very long, stout corona lobes carrying transverse rows of robust setae along their length, while *Stephanoceros millsii* has five relatively slender corona lobes carrying longitudinal rows of long, fine setae. The corona lobes of *Stephanoceros fimbriatus* are relatively shorter than those of *Stephanoceros millsii*, when compared to their trunk length. Regarding trophi, the unci tips of *Stephanoceros millsii* are acutely pointed whereas those of *Stephanoceros fimbriatus* have arrow-shaped tips, and the unci are more strongly curved in *Stephanoceros millsii* (compare Fig. 5F with Fig. 1B in Sørensen and Giribet 2006).

Because the morphological characters of these two taxa enable a reliable diagnosis and because the two have wide and overlapping distribution ranges, we argue that these two taxa are distinct species, in contrast to Koste (1978) who considered *Stephanoceros millsii* an infrasubspecific variant (“Anscheinend Ökotyp”) of *Stephanoceros fimbriatus*. Ours are the first photographs of living animals and trophi of *Stephanoceros millsii*.

*Stephanoceros millsii* is common in Thailand whereas *Stephanoceros fimbriatus* is quite rare in our survey. Both species are cosmopolitan (Koste 1978).

### The uncinate trophi of Collothecidae

The uncinate trophi type is one of nine trophi types recognized in phylum Rotifera (Wallace et al. 2006). This trophi type is characterised by unci possessing few teeth and by weakly developed manubria and fulcrum (Koste 1978) and has hardly been considered in the taxonomic analysis of Collothecacea (Families Collothecidae and Atrochidae). We examined the uncinate trophi of 6 species of Collothecidae to evaluate whether morphological differences, which might be taxonomically relevant, exist.

We found that, in all species examined, the uncinate trophi are composed of two pairs of large and sturdy unci teeth, whereas manubria, rami and fulcrum are less developed components (Figs 5A–F). Of the unci, the distal tips can be gradually sharpened (5C–D), stout (5B), or with set-off tips (5A), and the tips may carry a terminal, median groove (e.g., 5E–F). The unci are mostly strongly curved, either more or less evenly (e.g., 5A, E) or in their proximal third (5B), or terminally (5C), and the terminal tips may be slightly incurved (5A), straight (5E), or outcurved (5B). The unci pairs can be relatively equal (5A, D–F) or strongly unequal (5B, C) in length. The unci teeth are quite sturdy, as they are not easily dissolved by low concentration of commercial bleach (lower than 5% final concentration). The manubria, rami and fulcrum, on the other hand, are very weak and dissolve easily in bleach making it particularly hard to reliably compare their morphology. Nevertheless, the rami scleropilli usually remain after treatment (5E–F).

As illustrated here, the uncinate trophi, in particular the unci, do exhibit features that might be useful for taxonomic analysis. We suggest that 1) shape of the head of the unci; 2) shape of the unci teeth; and 3) relative size of the two pairs of unci teeth might be registered in future studies of *Collotheca* rotifers. Of course, the inclusion of these features in taxonomic analysis requires addition of information on more species of *Collotheca*, and evaluation of the intraspecific variability by comparing different populations of *Collotheca* species.

### Feeding in *Collotheca*

As mentioned above, Collothecidae species are essentially ambush predators. They remain immobile until a prey organism, guided by their long cilia and infundibulum that forms a fyke, and water current created by the beating of short cilia, comes in range of a sensory organ situated dorsally on the inner side of the infundibulum. When this organ is triggered, the cilia, corona lobes and infundibulum contract which restrains the prey organism within the infundibulum, and the prey is finally ingested whole. We observed that some species of *Collotheca*, and these appear to be species that have an enlarged funnel-shaped infundibulum, arrange their corona near the surface of the substrate they are attached to (e.g., *Collotheca* sp., Figs 3D; *Collotheca ferox*, Fig. 2A, B - note that the specimen in Figs 2A, B was not in normal position; *Collotheca orchidacea* sp. n., Figs 2C, D). Other species, mostly those that have a relatively smaller infundibulum but well-developed bands of cilia along the corona or on knobs, and a long foot, expose their expanded corona in the water column (e.g., *Collotheca campanulata* f. *longicaudata*, Fig. 3B; *Collotheca ornata*, Fig. 3C; *Collotheca tenuilobata*, Fig. 3F). We hypothesize that the two groups may have different diets. The latter group probably feeds on free-swimming, planktonic/periphytic organisms, while species of the former group may target browsing animals, in a way that is strikingly similar to *Cupelopagis vorax* (Leidy, 1857) (Bevington et al. 1995).

### Identification key to the Collothecidae of Southeast Asia

The keys presented here include all species recorded hitherto from Southeast Asia (Brunei, Cambodia, Indonesia, Malaysia, Myanmar, Philippines, Singapore, Thailand, Timor Leste, Vietnam), as included in De Ridder and Segers (1997) and more recent publications. To facilitate identification and discovery of species not included in the key, we provide both a dichotomous as well as a formula key to the Southeast Asian Collothecidae.

**Dichotomous key**

**Table:** 

1	Length of corona lobe(s) shorter than trunk (Figs 3A–F)	(genus *Collotheca*), 2
–	Length of corona lobes in adult specimens as long as, or longer than trunk (Figs 3G, H)	(genus *Stephanoceros*), 16
2(1)	Animals free-living (planktonic)	3
–	Animals fixosessile, permanently attached to a substratum	5
3(2)	Corona edge circular, smooth; inner side of infundibulum with five rudimentary lobes	*Collotheca pelagica*
–	Corona with projections bearing groups of cilia	4
4(3)	Corona with a single dorsal lobe carrying one group of long cilia	*Collotheca libera*
–	Corona with five knob-shaped projections, the dorsal one on a triangular lobe; all bearing a group of long cilia (Fig. 3C, 4B)	*Collotheca ornata* f. *natans*
5(2)	Corona with well-defined, rounded or club-shaped knobs (Figs 3C, F)	6
–	Corona circular or with broad lobes, no knob(s) (Figs 3B, D, E)	8
6(5)	Corona with seven knobs, the dorsal on a small, triangular lobe (Fig. 4A)	*Collotheca heptabrachiata*
–	Corona five projections (Fig. 4B)	7
7(6)	Corona with equal, elongated lobes terminating in club-shaped knobs (Fig. 3F)	*Collotheca tenuilobata*
–	Corona lobes unequal and/or less than three times their width (Figs 3C, 4B)	*Collotheca ornata*
	Within this species two infrasubspecific variants have been recorded from Southeast Asia. One (*Collotheca ornata* f. *natans*) is pelagic (see (3)), while *Collotheca ornata* f. *cornuta* is diagnosed by the presence of an elongate projection on the dorsal corona lobe (Figs 4G, H).
8(5)	Corona circular, smooth, bearing only short cilia	*Collotheca edentata*
–	Corona with broad lobes	9
9(8)	Corona with one large dorsal and one smaller ventral lobe, dorsal lobe with a group of elongate, parallel cilia (Fig. 3D)	*Collotheca* sp.
–	Corona with a dorsal lobe and a ventral sinus (Fig. 3B)	10
10(9)	Corona with three lobes separated by clear, smoothly concave sinuses between the dorsal and ventral lobes (Fig. 3E)	11
–	Corona with five lobes, the lateral ones may be only indicated (Figs 3B, 4F)	12
11(10)	Corona consisting of homogeneous rows of cilia (Fig. 3E)	*Collotheca trilobata*
–	Corona consisting of transversal sets of short, stiff cilia (Fig. 4D)	*Collotheca stephanochaeta*
12(9)	Lateral corona lobes larger than ventral lobes, these set close together and separated by a shallow and narrow V-shaped sinus (Figs 2A, B, 4J)	*Collotheca ferox*
–	Lateral corona lobes smaller than ventral lobes (Figs 3B, 4F)	13
13(12)	Lateral corona lobes well-develloped, thumb-shaped; ventral lobes large, rounded (Figs 2C–F)	*Collotheca orchidacea* sp. n.
–	Lateral corona lobes lower than wide or only indicated (Figs 3B, 4F)	14
14(13)	Ventral sinus deep, broadly U-shaped, wider than the width of the ventral lobes (Fig. 4E)	*Collotheca ambigua*
–	Ventral sinus shallow (Fig. 4F)	15
15(14)	Ventral lobes triangular with rounded tip, dorsal lobe relatively narrow (Fig. 4F)	*Collotheca algicola*
–	Ventral lobes rounded, dorsal lobe broad (Fig. 3B)	*Collotheca campanulata*
	Within this species one infrasubspecific variant has been recorded from Southeast Asia. *Collotheca campanulata* f. *longicaudata* is characterised by the presence of an extraordinary long peduncle (secreted attachment stalk: Fig. 4I).
16(1)	Corona lobes stout and robust, carrying parallel, transversal sets of robust cilia (Figs 3H, 4C)	*Stephanoceros fimbriatus*
–	Corona lobes slender, carrying dense, longitudinal rows of fine, cilia (Fig. 3G)	*Stephanoceros millsii*

### Formula key

**Characters**

1. Species (a) free-living (pelagic); (b) living attached to a substratum (fixosessile)
2. Corona edge: (a) circular, smooth; (b) with well-defined knobs (Figs 3C, F); (c) with lobes (Figs 3B, E)
3. Number of corona projections: (a) one dorsal; (b) two: one dorsal, one ventral (Fig. 3D); (c) three: one dorsal, two lateral (Fig. 3E); (d) five: one dorsal, two lateral, two ventral (Figs 3B, 4F); (f) seven (Fig. 4A)
4. Length of corona projections: (a) much shorter than trunk (Figs 3C, D); (b) strongly elongated and parallel sided (Figs 3G, H)
5. Diversification of corona projections: (a) none, all projections more or less equal (Figs 3A, F–H); (b) differentiated (Figs 3B–E)
6. Lateral corona lobes: (a) absent (3E); (b) indicated (sinus between dorsal and ventral lobe is not smoothly concave or indicated by presence of a distinct group of particularly long cilae: Figs 4E, F); (c) well-developed (Figs 2A–F)
7. Ventral corona projections: (a) with one midventral lobe, (b) with two knobs (Fig. 4B); (c) two rounded triangular lobes (Fig. 4F); (d) two semicircular lobes (Figs 3B, E)
8. Ventral corona sinus: (a) shallow, narrow (Fig. 4J); (b) shallow, broad (Figs 3B, 4F); (c) deep, broad, U-shaped (Fig. 4E)
9. Special features: (a) elongate projection dorsally on dorsal corona lobe (Figs 4G, H); (b) peduncle (attachment stalk) longer three times diameter of foot than foot (Fig. 4I); (c) cilia inserted in parallel sets of transverse rows (Figs 4C, D); (e) group of elongate cilia dorsally on dorsal corona lobe (Fig. 3D)

### Species

*Collotheca algicola*: 1b, 2c, 3d, 4a, 5b, 6b, 7c, 8b

*Collotheca ambigua*: 1b, 2c, 3d, 4a, 5b, 6b, 7d, 8c

*Collotheca campanulata*: 1b, 2c, 3d, 4a, 5b, 6b, 7d, 8b (+9b: f. *longicaudata*)

*Collotheca edentata*: 1b, 2a

*Collotheca ferox*: 1b, 2c, 3d, 4a, 5b, 6c, 7c, 8a

*Collotheca heptabrachiata*: 1b, 2b, 2c, 3f, 4a, 5b, 6a, 7b

*Collotheca libera*: 1a, 2c, 3a, 4a

*Collotheca orchidacea* sp. n.: 1b, 2c, 3d, 4a, 5b, 6c, 7d, 8c

*Collotheca ornata*: (1b), 2b, 2c, 3d, 4a, 5b, 6b, 7b (+9a: f. *cornuta*; 1a: f. *natans*)

*Collotheca pelagica*: 1a, 2a

*Collotheca stephanochaeta*: 1b, 2c, 3c, 4a, 5a, (6b), 7d, 8b, 9c

*Collotheca tenuilobata*: 1b, 2b, 2c, 3d, 4b, 5a

*Collotheca trilobata*: 1b, 2c, 3c, 4a, 5b, 6a, 7d, 8c

*Collotheca* sp.: 1b, 2c, 3b, 4a, 5b, 6a, 7a, 9e

*Stephanoceros fimbriatus*: 1b, 2c, 3d, 4b, 5a, 9c

*Stephanoceros millsii*: 1b, 2c, 3d, 4b, 5a

## Supplementary Material

XML Treatment for Collotheca ferox

XML Treatment for Collotheca orchidacea

XML Treatment for Collotheca ornata f. cornuta

XML Treatment for Collotheca

XML Treatment for Stephanoceros

## References

[B1] Bevington DJ White C Wallace RL (1995) Predatory behavior of *Cupelopagis vorax* (Rotifera; Collothecacea; Atrochidae) on protozoan prey. Hydrobiologia 313/314: 213–217. doi: 10.1007/BF00025953

[B2] Chittapun S Pholpunthin P Segers H (2007) Diversity of rotifer fauna from five coastal peat swamps on Phuket island, southern Thailand. ScienceAsia 33: 383-387. doi: 10.2306/scienceasia1513-1874.2007.33.383

[B3] De Ridder M Segers H (1997) Rotifera Monogononta in six zoogeographical regions after publications between 1960–1992. Studiedocumenten van het Koninklijk Belgisch Instituut voor Natuurwetenschappen 88: 1-481.

[B4] Edmondson WT (1940) The Sessile Rotatoria of Wisconsin. Transactions of the American Microscopical Society 59: 433-459. doi: 10.2307/3222991

[B5] Kellicott DS (1887) Additional notes on certain species of Rotifera. Proceedings of the American Society of Microscopists 9: 181-186. doi: 10.2307/3220550

[B6] Koste W (1975) Über den Rotatorienbestand einer Mikrobiozönose in einem tropischen aquatischen Saumbiotop, der *Eichhornia-crassipes*-Zone im Litoral des Bung-Borapet, einem Stausee in Zentralthailand. Gewässer und Abwässer, 57/58:43–58.

[B7] Koste W (1978) Rotatoria. Die Rädertiere Mitteleuropas. Borntraeger, Berlin, 2 vols, 673 pp, 234 plates.

[B8] Kutikova LA Markevich GI (1993) Principal directions of the evolution of Monimotrochida. Hydrobiologia255/256: 545–549. doi: 10.1007/BF00025883

[B9] Meksuwan P Pholpunthin P Segers H (2011) Diversity of sessile rotifers (Gnesiotrocha, Monogononta, Rotifera) in Thale Noi Lake, Thailand. Zootaxa 2997: 1-18.

[B10] Penard E (1914) A propos de Rotifères. Revue Suisse de Zoologie 22: 1-27.

[B11] Sanoamuang L Savatenalinton S (2001) The rotifer fauna of Lake Kud-Thing, a shallow lake in Nong Khai Province, northeast Thailand. Hydrobiologia 446/447: 297–304. doi: 10.1023/A:1017588331347

[B12] Segers H (1993) Rotifera of some lakes in the floodplain of the River Niger (Imo State, Nigeria). I. New species and other taxonomic considerations. Hydrobiologia 250: 39-61. doi: 10.1007/BF00007494

[B13] Segers H (2002) The nomenclature of the Rotifera: annotated checklist of valid family- and genus-group names. Journal of Natural History 36: 631-640. doi: 10.1080/002229302317339707

[B14] Segers H (2007) Annotated checklist of the rotifers (Phylum Rotifera), with notes on nomenclature, taxonomy and distribution. Zootaxa 1564: 1-104.

[B15] Segers H Chittapun S (2001) The interstitial Rotifera of a tropical freshwater peat swamp on Phuket Island, Thailand. Belgian Journal of Zoology 131 (Supplement 2): 65–71.

[B16] Segers H Sanoamuang L (2007) Note on a Highly Diverse Rotifer Assemblage (Rotifera: Monogononta) in a Laotian Rice Paddy and Adjacent Pond. International Review of Hydrobiology 92: 640-646. doi: 10.1002/iroh.200610968

[B17] Segers H Meksuwan P Sanoamuang L (2010) New records of sessile rotifers (Rotifera: Flosculariacea, Collothecacea) from Southeast Asia. Belgian Journal of Zoology 140 (2): 235-240.

[B18] Sørensen MV Giribet G (2006) A modern approach to rotiferan phylogeny: Combining morphological and molecular data. Molecular Phylogenetics and Evolution 40: 585-608. doi: 10.1016/j.ympev.2006.04.001 16690327

[B19] Wallace RL Snell TW Ricci C Nogrady T (2006) Rotifera vol 1: Biology, Ecology and Systematics. In: Segers H, Dumont HJF (Eds) Guides to the Identification of the Microinvertebrates of the Continental Waters of the World, 23, Kenobi productions, Ghent, Belgium and Backhuys Academic Publishing bv, The Hague, The Netherlands, 299 pp.

